# Deep Learning-Based Image Classification for Major Mosquito Species Inhabiting Korea

**DOI:** 10.3390/insects14060526

**Published:** 2023-06-05

**Authors:** Sangjun Lee, Hangi Kim, Byoung-Kwan Cho

**Affiliations:** 1 Department of Biosystems Machinery Engineering, Chungnam National University, Daejeon 34134, Republic of Korea; sangjoon10005@naver.com (S.L.); zxcvkhk@gmail.com (H.K.); 2 Department of Smart Agricultural System, Chungnam National University, Daejeon 34134, Republic of Korea

**Keywords:** mosquito, artificial intelligence, deep learning, image identification

## Abstract

**Simple Summary:**

Conventional manual counting methods for the monitoring of mosquito species and populations can hinder the accurate determination of the optimal timing for pest control in the field. In this study, a deep learning-based automated image analysis method was developed for the classification of eleven species of mosquito. The combination of color and fluorescence images enhanced the performance for live mosquito classification. The classification result of a 97.1% F1-score has demonstrated the potential of using an automatic measurement of mosquito species and populations in the field. The proposed technique could be adapted for establishing a mosquito monitoring and management system, which may contribute to preemptive quarantine and a reduction in the exposure to vector-borne diseases.

**Abstract:**

Mosquitoes are one of the deadliest insects, causing harm to humans worldwide. Preemptive prevention and forecasting are important to prevent mosquito-borne diseases. However, current mosquito identification is mostly conducted manually, which consumes time, wastes labor, and causes human error. In this study, we developed an automatic image analysis method to identify mosquito species using a deep learning-based object detection technique. Color and fluorescence images of live mosquitoes were acquired using a mosquito capture device and were used to develop a deep learning-based object detection model. Among the deep learning-based object identification models, the combination of a swine transformer and a faster region-convolutional neural network model demonstrated the best performance, with a 91.7% F1-score. This indicates that the proposed automatic identification method can be rapidly applied for efficient analysis of species and populations of vector-borne mosquitoes with reduced labor in the field.

## 1. Introduction

Vector-borne diseases account for more than 17% of all infectious diseases, affect millions of people, and cause more than 700,000 deaths annually [1]. The World Health Organization (WHO) has reported that mosquitoes are the deadliest insects in the world and infect humans with various diseases that are fatal. As global temperatures rise, the mosquito population will increase owing to the characteristics affected by temperature [2]. To prevent mosquito-related diseases, it is necessary to identify and predict mosquito distribution. The current process of identifying species and populations is manual, labor-intensive, time-consuming, and requires expert knowledge. In addition, humans make identification errors at times, increasing the wastage of human labor and material resources, such as insecticides [3].

Climatic conditions such as temperature and precipitation influence the occurrence of Japanese encephalitis, malaria, dengue fever, and West Nile vector disease [4,5,6,7]. This increases the density of vector-borne diseases, and the high disease density may lead to an epidemic. Korea has five mosquito-borne infectious diseases: malaria, Japanese encephalitis, Zika fever, yellow fever, and West Nile fever. With a rise in temperatures, the population of *Culex tritaeniorhynchus Giles, 1901* (*Cx.tri*), the vector of Japanese encephalitis, is especially increasing in Korea [8]. Among the many mosquito-borne diseases, malaria, dengue, and yellow fever affect more than one million people annually [1]. There were an estimated 241 million infection cases worldwide of vector-borne diseases, such as malaria, in 2020 [9]; approximately 3 billion people in Southeast Asia and the Western Pacific region are at risk of being exposed to Japanese encephalitis [10]; infections due to dengue are estimated at 100 to 400 million [11]; West Nile infections totaled 737, and the corresponding deaths totaled 79 in the USA [12]; Zika virus infections totaled 31,888, and deaths totaled 4 in the Americas and the Caribbean [13]. Malaria and Japanese encephalitis are representative vector-borne diseases in Republic of Korea [14]. Dengue, chikungunya, and Zika viruses have no cases of indigenous infection in Republic of Korea; however, vector-borne mosquitoes exist in Republic of Korea. Therefore, if cases of inflows through overseas infections increase, infectious diseases can be transmitted, and dengue can be spread through indigenous mosquitoes [15].

Machine learning and deep learning have been used to identify mosquitoes and prevent the spread of vector-borne diseases. Park et al. studied *Culex pipiens pallens Coquillett*, *1898* (*Cx. pip*), *Aedes albopictus Skuse, 1895* (*Ae. albo*), *Anopheles* spp., and other flying insects by applying mosquito population information obtained from a digital mosquito monitoring system (DMS) to a ResNet-based faster region-convolutional neural network (R-CNN) [16,17]. To identify the species of mosquitoes using the sounds of mosquito wings, studies have suggested species classification models based on machine learning classifiers using wavelet transformation and the collected acoustic resources of mosquito wings [18,19]. Goodwin et al. [20] constructed an algorithm that utilized the Xception model to identify unlearned species. Siddiqua et al. [21] used Inception V2 and faster R-CNN to detect dengue. To detect *Aedes aegypti Linnaeus, 1762* and *Aedes albopictus*, a mosquito classification and detection technique was developed using AlexNet and a support vector machine (SVM), and the features of each body part were extracted [22,23]. Despite the attempts to develop various mosquito detection and classification models, most of them involved image classification rather than object detection, or the results for similar species were not specified. Therefore, existing research is considered unsuitable for real-field applications owing to the use of mosquito corpses. However, there are a few studies on living and similar species.

**Figure 1:**
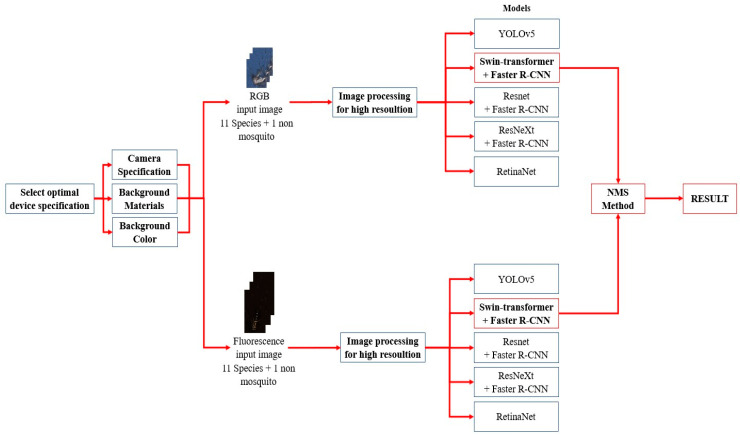
Flow chart of this research. It comprises three main parts: image preprocessing, prediction, and NMS method. Image preprocessing improve the image quality. Deep learning model predicts each type of images. The two types of images are combined to improve the accuracy.

In this study, we propose an automatic mosquito classification technique for identifying vector-borne mosquitoes in Republic of Korea. To develop this technique, a deep learning detection model was combined with Faster R-CNN [17,21,24,25,26] and Swin Transformer [27], which demonstrate the highest performance in object detection among the models. You Only Look Once (YOLO) v5 [28], ResNet DC-5 [29], ResNeXt 101 [30], and RetinaNet [31] were used to compare the performances of the mosquito detection models. Color and fluorescence images of the mosquitoes were used to extract various features. To improve the model identification performance, non-maximum suppression (NMS) was applied by combining two types of images. A combination of color and fluorescence images was used to enhance detection accuracy. The entire procedure was summarized in Figure 1. If automatic mosquito species classification techniques are developed, labor and resource costs can be reduced, and pesticides can be applied in an accurate and timely manner to target species, reducing the time and the unnecessary waste of resources for pest control operations.

The remainder of this study is structured as follows: Section 2 describes the datasets, background material comparisons, models, and methods used in this study. Section 3 describes the materials, efficiency of the methods, and performance of the models. Section 4 discusses conclusions.

## 2. Materials and Methods

### 2.1. Sample Collection and Features

**Figure 2:**
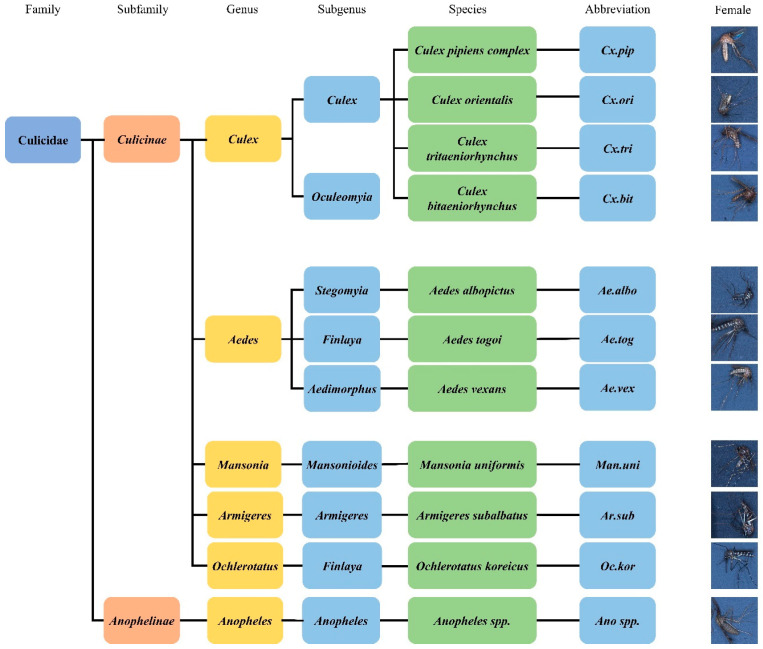
Classification and abbreviation of the mosquitoes used in training.

We used 11 species of mosquitoes and non-mosquito (*Chironomus*), totaling 12 classes as shown in Figure 2. According to the Korea Disease Control and Prevention Agency (KDCA), six species of mosquitoes can breed: *Culex pipiens complex*, *Culex tritaeniorhynchus summorosus*, *Aedes albopictus*, *Aedes vexans nipponii Theobald*, *1907* (*Ae. vex*), *Anopheles* spp. (*An.* spp.), and *Aedes togoi Theobald*, *1907* (*Ae. tog*). However, the remaining species have difficulty breeding. These species were collected from wild environments. The number of non-breeding mosquitoes was smaller than that of breeding species because of the difficulty in collecting them from the wild environment. Alive mosquitoes were placed in a prototype device using an aspirator to train the mosquitoes similarly to conditions in the real field.

Several different species of mosquitoes were captured using a capturing device, and their images were obtained. For deep learning training, the individual mosquito images were cropped from the 1677 respective color and fluorescence images. The detailed information of individual mosquito image depending on the species are indicated in Table 1. The dataset was divided according to a 7:2:1 ratio, with the training set for training, the validation set for evaluating the performance, and the test set for testing the classification accuracy of the model. Part of the training set was allocated as a validation set, which had the advantage of predicting the accuracy of the test set and preventing overfitting. Owing to the small populations of *Culex bitaeniorhynchus Giles*, *1901* (*Cx. bit*), *Culex orientalis Edwards*, *1921* (*Cx. ori*), and *Chironomus*, the ratios in the validation and test sets were the same. Image labeling was performed using COCO Annotator [32], a web-based labeling tool.

**Figure 3:**
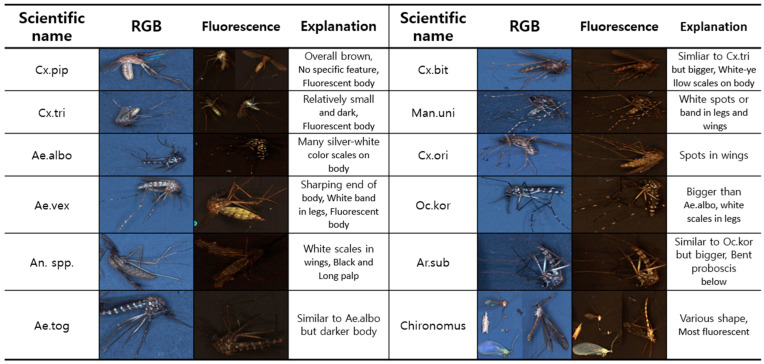
Characteristics of mosquitoes.

Mosquitoes have diverse characteristics as shown in Figure 3. Morphological and entomological taxonomic characteristics were obtained from the electronic mosquito encyclopedia of the KDCA. *Cx.pip* has an overall brown color and no specific feature. It exhibits fluorescence. *Cx.tri* is similar to *Cx.pip* but is relatively small and dark. In addition, it exhibits fluorescence. *Ae.albo*, *Ae.tog*, *Ochlerotatus koreicus Edwards*, *1917* (*Oc. kor*), and *Armigeres subalbatus Coquillet*, *1898* (*Ar. sub*) appear similar but have different characteristics. *Ae.albo* has many silver-white scales on its body. *Ae.tog* is similar to *Ae.albo* but darker. *Oc.kor* is larger than *Ae.albo* and has white scales on its legs. *Ar.sub* is similar to *Oc.kor* but bigger and has a stooped proboscis. *Ae.vex* has a sharp body end, white bands on the legs, and a fluorescent body. It is easy to distinguish *An.* spp. from other mosquitoes because of their unique characteristics. It has white scales on its wings, with long black palps. *Cx.bit* is similar to *Cx.tri* but has larger white-yellow scales on the body. *Mansonia uniformis Theobald*, *1901* (*Man. uni*) has distinct white spots or bands on its legs and spots on its wings. *Cx.ori* has spots on its wings. Furthermore, it is difficult for non-experts in entomological taxonomy to classify mosquitoes as vector-borne because of their various characteristics. Therefore, a model for the automatic classification of mosquitoes can reduce human labor, classification time, and human error.

### 2.2. Data Collection

Figure 3 demonstrates various features, such as mosquito patterns, colors, and shapes. The image quality affects the model performance. To improve the model performance, three conditions were considered for capturing high-quality images: camera specifications, illumination, and background.

#### 2.2.1. Camera Specification

Because the average size of a mosquito is approximately 15 mm, it may be difficult to detect various features (body parts and patterns) without a high resolution, resulting in detection errors [33,34]. Therefore, high-resolution cameras are required to detect objects. A 20 MP color camera (mvBlueFOX3-2205C-2212, SONY IMX183, Matrix Vision GmbH, Oppenweiler, Germany) with a C-mount lens (V5024-MPZ, CBC AMERICA, Cary, NC, USA) was used. The camera resolution was determined by considering the pixels per inch (PPI), mosquito size, and field of view (FOV, 90 × 70 mm) to obtain sufficient resolution for the object. In addition, the camera could be easily applied to the actual field and remotely controlled, as it could be controlled by embedded systems such as Jetson AGX Xavier [35] and Linux.

#### 2.2.2. Illumination

**Figure 4:**
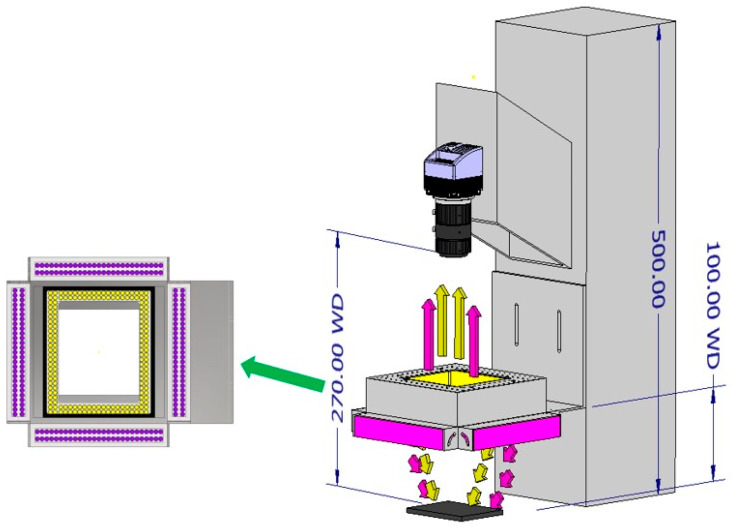
Image capturing prototype. White and UV LED were set to collect two types of images.

Low-power, long-life white LED and UV LED were used for illumination by considering the characteristics of devices mainly operated during the summer [36]. Color images with white LED and fluorescence images with UV LED could provide both the morphological and fluorescence features of mosquitoes, which could enhance the classification accuracy of mosquitoes. An image-capture architecture was constructed by combining a camera and illumination to obtain RGB and fluorescence images. A single white LED was located at the center, and four UV LEDs were placed outside the white LED. The UV LED was focused on the background at an angle of 45°, as shown in Figure 4. The camera settings are listed in Table 2. The distances between the camera, light, and background panels were determined by considering the working distance (WD) and FOV. The image-capturing process was as follows: first, the white LED was turned on, and a color image of the mosquitoes was captured. Thereafter, the white LED was turned off. Next, the UV LED was turned on, and the fluorescence image of the mosquitoes was captured. The process was controlled using Python software.

#### 2.2.3. Background Color and Materials

Background color and materials affect the image quality. Black, white, and blue were selected to evaluate the most improved colors in the images. Blue was selected because it does not exist in the environment. Black paper, two types of polyvinyl chloride (PVC), and steel panels were used as background materials to improve image quality.

### 2.3. Image Preprocessing

When the mosquitoes entered the capturing device, they were subjected to electric shock. They randomly fell into the background at various positions. Most of them fell sideways or displayed bellies. In some cases, the legs or proboscis were outside the depth of the camera. The optimal depth was selected by adjusting the aperture because the depth decreases as the camera’s aperture expands [37]. Technologies such as sharpening, brightness adjustment, and histogram stretching were applied to improve the quality of the color and fluorescence images. A Python OpenCV image processing sharpening method was applied to improve the image quality and extract clear features of the wings and patterns [38]. Sharpening is an image-filtering technique that increases the contrast ratio at the edge of an object where pixel values change rapidly. In the fluorescence image, it was difficult to recognize the features of the scales of the wings and patterns of the body owing to their darkness. Therefore, the brightness, histogram stretching, and sharpening techniques of OpenCV were applied to improve the image quality. Brightness adjustment using an additive operation on the pixels brightens images [39]. If the image is bright, its contrast ratio is low. The distinction between objects was unclear [40]. After brightness adjustment, histogram stretching was performed to increase the contrast ratio between the mosquito and background images [41]. After adjusting the contrast ratio, the image quality was improved using a sharpening technique.

### 2.4. Deep Learning

Most image object detection methods are based on CNNs [42] because a CNN is a neural network model that overcomes the deep neural network (DNN), which uses one-dimensional data and can extract features without the loss of spatial and regional image information. Starting with CNN, various models have continued to develop, including two types of models: a one-stage detector, which is fast but less accurate, such as YOLO [43], a single-shot multibox detector (SDD) [44], and a two-stage detector, which is slow but more accurate, such as R-CNN [45], Fast R-CNN [46], and Faster R-CNN [26]. In this study, we focused on the accuracy of the model. Therefore, we constructed a two-stage object detection model using Detectron2 [47]. A Detectron2 developed by the Meta Company provides the latest detection and segmentation algorithms, deep learning API, and various pretrained weights [47]. Furthermore, we used five models: Faster R-CNN with Swint-transformer, YOLOv5, ResNet101 DC-5, ResNeXt 101, and RetinaNet.

### 2.5. Models

#### 2.5.1. YOLOv5

YOLOv5 has a backbone based on CSPNet. The advantage of CSPNet is that it reduces the amount of computation required to create gradient combinations [48]. Therefore, YOLOv5 decreases the memory cost and inference time and improves the model accuracy [28]. YOLOv5 provides various versions of a model by controlling the depth and width of the feature extractor. YOLOv5 also utilizes an FPN, which evolves a model to detect different scales of objects using a hierarchical structure to improve accuracy [28].

#### 2.5.2. Faster R-CNN with Swin-Transformer

The models were based on Detectron2, except YOLOv5. We used a Detectron2 Faster R-CNN with a swin transformer. The faster R-CNN model was highly accurate in Detectron2 and was used as a detector for the object detection model. Swin transformers exhibit excellent accuracy in object detection. Additionally, that model is a state-of-the-art model that has evolved from transformers used in natural language processing (NLP) to the computer vision domain. It also has a hierarchical structure and requires fewer computations than other methods. Thus, it can extract various scales of objects, enlarge the model size, and obtain a high inference speed. It has achieved high accuracy compared to previous models based on common datasets such as ImageNet [49], COCO [50], and ADE20K [51].

#### 2.5.3. ResNet DC-5 and ResNeXt 101

Backpropagation was used to correct the parameters of the weights in the deep learning training process. However, a gradient vanishing problem was observed owing to the successive multiplication of the derivative. ResNet solves this problem using a shortcut connection. The input value x was added to the output value after a few layers to solve the vanishing gradient [52]. Consequently, more deep learning layers could be stacked, which enabled the models to achieve higher accuracy.

ResNet101 DC-5 was developed using a deformable ConvNet. DC-5 implies that dilation is added to conv5 to increase the feature resolution of the backbone [29]. ResNeXt is an evolved model of ResNet that applies the cardinality method. Cardinality is a hyperparameter that divides the channel size into groups. Each channel focuses on different features and adds them to a subsequent layer. Therefore, it can be used to train various features. This shows that increasing the cardinality is more effective than enlarging the channel and depth size [30].

#### 2.5.4. RetinaNet

RetinaNet is a one-stage detector that addresses the class imbalance problem between the foreground and background. Researchers proposed a novel focal loss function. The focal loss adjusts the dynamic scaling factor, which changes based on the cross-entropy loss. The loss function can focus on training by increasing the weights of the hard examples and decreasing those of the easy examples [31].

### 2.6. Hyperparameter Settings

The datasets contained approximately 7000 mosquitoes. Furthermore, extensive performance analysis of models that extract features from mosquitoes to detect them was conducted. Based on memory limitations, learning speed, and generalization of performance, the hyperparameters were set as follows: batch, 32; learning rate, 0.0001; and weight decay, 0.05. The maximum number of iterations was set to 100,000 to collect sufficient results, and the validation set was evaluated every 500 iterations. Despite the maximum number of iterations being set to 100,000, if the validation loss did not decrease, we stopped early in the middle of the learning process.

### 2.7. Combining Prediction Results

**Figure 5:**
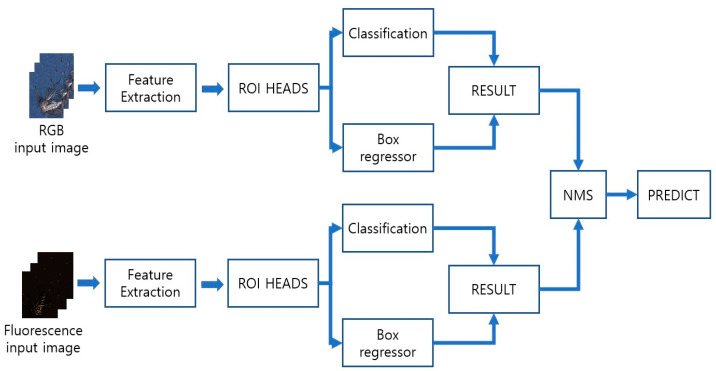
Flow chart of the NMS combining prediction. Predict the result in each image and use the NMS method. This method achieves higher accuracy then the other.

Simple learning has a limitation in deriving optimal results. Therefore, a method to increase accuracy is required. The NMS was used to improve the identification accuracy of the model. This method suppressed all predictions except the one with the maximum accuracy. Based on the accuracy of the object-detection boundary box, the NMS lists the prediction boxes of the two types of images in order of high accuracy and compares the intersection over union (IoU) of the remaining bounding box with the maximum accuracy value. In addition, boxes above a certain threshold were removed [53]. In this study, the threshold was set to 0.5. Optimal results were derived using NMS, which combined color and fluorescence images. The accuracy of the prediction model could be improved using this method. Figure 5 presents a flowchart of the combined prediction.

### 2.8. Model Evaluation

Measurements such as precision, recall, F_1_-score, and accuracy were used to evaluate the model performance. These measurements use true positives, true negatives, false positives, and false negatives to measure performance.

(1) $$precision\;\left(P\right)=\frac{True\;Positive}{True\;Positive+False\;Positive}$$

(2) $$recall\;\left(R\right)=\frac{True\;Positive}{True\;Positive+False\;Negative}$$

(3) $$F_{1}\;score=2\times \frac{P\times R}{P+R}$$

## 3. Results and Discussion

### 3.1. Effect of Background Color and Materials on Image

**Figure 6:**
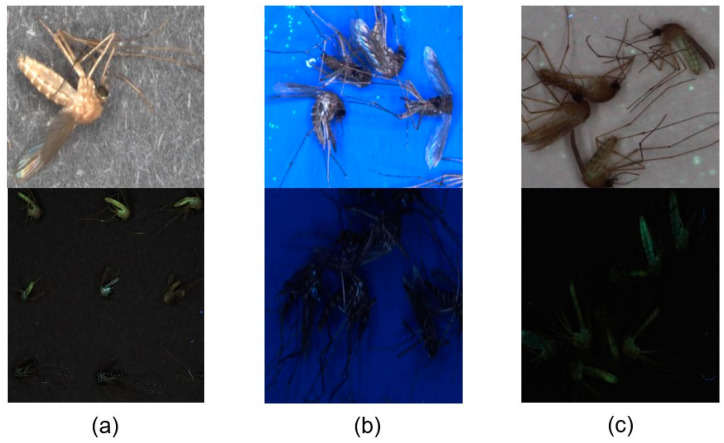
Background Color Comparison: (**a**) Black Background; (**b**) Blue Background; (**c**) White Background.

**Figure 7:**
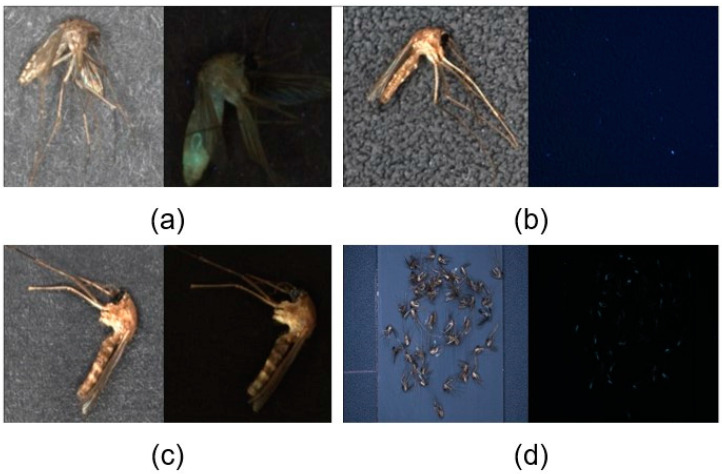
Background Materials Comparison: (**a**) Black Paper; (**b**) PVC (Interior Film); (**c**) PVC (Electric Tape); (**d**) Steel.

Among the black, white, and blue backgrounds, black was selected because it was of better quality than white and UV light, as shown in Figure 6. The blue background showed good image quality regarding color but not fluorescence. The white background had the opposite effect from that of blue. Black paper, two types of PVC, and a steel panel were used to compare the effects of background materials on the image quality, as shown in Figure 7. Black paper had white patterns in the background, which could be noise that disturbs feature extraction. The PVC (interior film) exhibited various irregular patterns that could be noise. The PVC (electric tape) did not have any specific patterns and was suitable for fluorescence imaging. However, fluorescent substances can disturb the feature extraction of fluorescence. The automatic mosquito-capturing architecture operates in summer, and it is sealed. Inner devices will be heated, but the tape is too weak to be heated. Therefore, steel was selected as the most suitable background material for this study.

### 3.2. NMS Method

**Figure 8:**
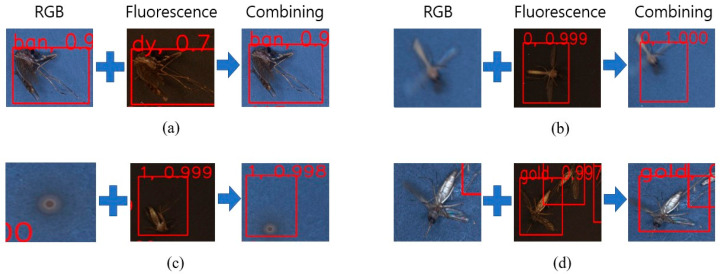
Examples of combining method: (**a**) Correct prediction; (**b**) Detection moving mosquito; (**c**) Detection rotating mosquito; (**d**) Prediction of not-detected mosquito.

As shown in Figure 8, the images on the left side of the arrow are RGB and fluorescent images before NMS application, while the images on the right side are obtained after NMS application. This method can improve the prediction performance more effectively than previous ones. The fluorescent features of mosquitoes could be used to supplement the color features.

The mosquito could not be detected because of movement in the RGB image, but it was predicted correctly because of a lack of movement in the fluorescent image. Figure 8 also shows the difficulty in detecting the mosquito owing to its rotation during movement. However, it was possible to predict using the fluorescent image that was captured correctly. Even if the mosquitoes move, shake, or fail to be predicted by RGB images, if the mosquitoes are correctly predicted in fluorescence images, it can be predicted. Higher classification accuracy was achieved by applying the NMS method. However, if the mosquitoes are captured well but have different coordinates in both types of images, the NMS method counts the mosquitoes twice. This might lead to counting errors.

### 3.3. Results of Models Using NMS

**Figure 9:**
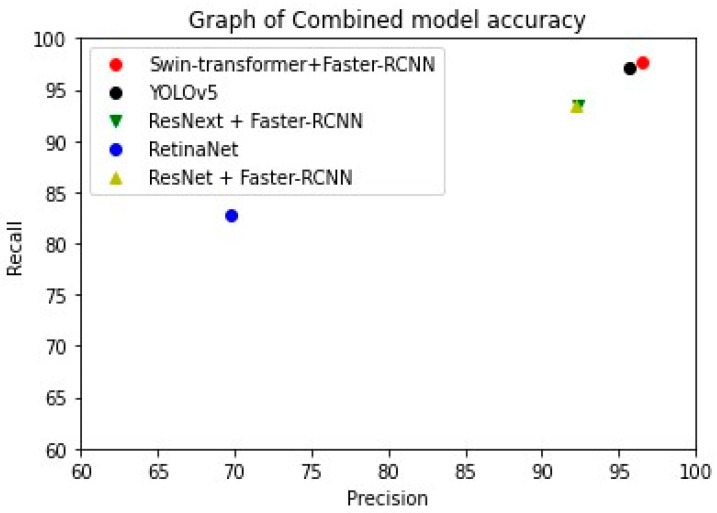
Graph of combined model accuracy. Swin-transformer and YOLOV5 have high top-2 accuracy.

Five types of models were used to evaluate the performance of the automatic mosquito-detection model. Figure 9 shows the results of the models. Table 3 presents the accuracy of the models. The swine transformer + Faster R-CNN model achieved the highest accuracy among the models. The accuracy of the model was the highest for the swine transformer at a 97.1% F1-score, followed by YOLOv5 at 96.5%. YOLOv5 has high accuracy, mostly in RGB images. This implies that YOLOv5 extracts features better in RGB images than in fluorescence images. Therefore, it does not generate significant improvements in accuracy. However, the swine transformer showed similar accuracy for RGB and fluorescence. This implies that different features were well extracted for both RGB and fluorescence. RetinaNet misidentified most mosquitoes as *Cx. pip. Cx. pip* does not have any specific features and is brown. The ResNeXt + Faster R-CNN model did not correctly predict many mosquitoes for *Cx. tri*, *Cx. pip*, and *Ae. tog*. Furthermore, they neither had any specific features nor extracted the correct features. ResNet also classified various mosquitoes as *Cx. tri* and *Cx. pip*. In the case of ResNet, the performance of the fluorescent images was higher than that of the RGB images. As shown in Table 4, applying the NMS methods increased the performance of all models. Therefore, considering these two factors, the fluorescence features of mosquitoes were different from those in RGB images. This could be another key classification method for identifying mosquitoes. Thus, utilizing the features extracted from fluorescence images with a swin transformer as the backbone and applying NMS methods can help improve the performance of the detection models. However, the detection of mosquitoes remains problematic. In general, most detection models were confused among *Cx. bit, Cx. ori*, and *Man. uni.* We believe that this is because of the small mosquito population. Therefore, it is considered that we need more mosquitoes.

### 3.4. Comparison of the Two Best Models

Swin-transformer + faster R-CNN and YOLOv5 achieved the two highest accuracy rates, as listed in Table 5. Both models exhibited sufficiently high accuracy for RGB and fluorescence measurements. Most mosquitoes were predicted more accurately using RGB than using fluorescence. We compared the prediction results of the two models for both RGB and fluorescence. Yolov5 had a higher accuracy in RGB images than the Swin-Transformer. However, the Swin-Transformer showed higher accuracy for *Cx. bit* than YOLOv5. YOLOv5 showed high accuracy, mostly for RGB images, whereas the swine transformer had a similar accuracy ratio between RGB and fluorescence. Fluorescence imaging can improve the performance of models in predicting the presence of mosquitoes. The RGB and fluorescence predictions were considered to be complementary. A combination of RGB and fluorescence may contribute to the development of a predictive model.

### 3.5. Discussion

Park et al. [54] constructed mosquito classification algorithms of eight species using VGG-16, Resnet-50, and SqueezeNet and achieved a classification accuracy of 97.19%. In addition, a classification accuracy of up to 80% was achieved as a result of prediction by putting two species of wild live mosquitoes (*Culex pipiens* and *Anopheles* spp.) as a test set. Siddiqua et al. [21] developed a dengue mosquito detection model using Inception V2 and Faster R-CNN and achieved a classification accuracy of 95.19%. Motta, D. et al. [55] also applied the convolutional neural network for classification of dengue mosquitoes using CNN models such as LeNet, AlexNet, and GoogleNet, and the classification accuracy was 76.2%. It presented the possibility to train the features of mosquitoes. Couret, J. et al. [56] delimited the variations of cryptic morphological characteristics using a CNN model. It achieved the classification accuracy of 96.96% for species identification and 98.48% for gender identification. Motta, D. et al. [57] constructed the classification model optimized hyperparameter for classifying to adult mosquitoes. The classification accuracy between mosquitoes and other insects achieved 93.5%, and the classification accuracy between Aedes and Culex achieved 97.3%. Zhao, De-zhong et al. [58] constructed mosquito classification algorithms of 17 species using Swin-transformer. Through comparison of several models and image size, the optimal model was selected. Additionally, a CNN was used in most papers [54,55,56,57,59,60] except Zhao, De-zhong et al. We achieved a 97.1% F1-score. It was not much higher than other paper’s achievement, but we have further advantages. In prior papers, most of the captured mosquitoes were taken in a laboratory environment for learning and evaluation. Even if alive mosquitoes were used as in the actual field, the number of species was small, or the accuracy was not high. In this study, images were taken after alive mosquitoes were used as train and evaluation data, so it is more suitable for field application. Additionally, mosquitoes and other insects were trained together so that it could decrease the number of cases which are confused by insects which are not mosquito. Fluorescence images were used to supplement the classification accuracy of color images. We improved classification accuracy by applying NMS as supplementary measures that need color and fluorescence images to be used.

## 4. Conclusions

In this study, we propose a model for the automatic identification of vector-borne mosquitoes that are deadly to humans. The dataset comprised 12 species, with 5 main vector-borne, 6 non-vector-borne, and 1 non-mosquito species (*Chironomus*). Live mosquitoes were used to collect datasets and capture images similar to those in the real field. Two types of images were obtained using two types of LEDs: white and UV LEDs. The image quality was improved by applying aperture control and computer vision pre-processing. The NMS method presented in this study was used to achieve the highest identification accuracy by combining the prediction results of the two types. Five detection models were selected, and their performances were compared. The swine transformer + Faster R-CNN had the best identification accuracy, with a F1-score of 97.1%. This automatic mosquito capture architecture can be used to detect mosquito species and populations in the field. It can also predict the generation of mosquitoes, such that preemptive measures could be performed more rapidly than before. This can reduce human labor, time consumption, and resources for identifying mosquitoes.

However, owing to the small size of the datasets, a few species lacked populations. Some mosquito species are not robust against various features. In the NMS method, one mosquito was counted twice when it was moving and was not stunned. In future studies, we will collect more mosquitoes that appear to be similar to the five main species. To decrease errors such as missed counting, we will change the stunning or counting process of the NMS method.

## Figures and Tables

**Table 1:** Datasets for mosquito identification model training.

Species	Train	Validation	Test	Total
*Cx. pip*	830	158	56	1044
*Cx. tri*	1022	193	117	1332
*Ae. albo*	592	127	68	787
*Ae. tog*	381	102	52	535
*Ae. vex*	551	278	44	873
*An.* spp.	634	165	94	893
*Cx. bit*	118	50	50	218
*Cx. ori*	90	39	39	168
*Man. uni*	171	53	33	257
*Oc. kor*	251	84	47	382
*Ar. sub*	182	60	28	270
*Chironomus*	157	90	90	337
Total	4979	1339	718	7096

**Table 2:** Camera setting values.

LEDs	Exposure Time (μm)	Gain	White Balance (Red)	White Balance (Blue)	Gamma
White LED	80,000	0.0	167.0	223.0	0.8
UV LED	200,000	27.0	267.0	91.0	0.8

**Table 3:** Evaluation of detection models.

Models	RGB			Flourescence		
	Precision	Recall	F1-Score	Precision	Recall	F1-Score
Swin-transformer + Faster R-CNN	93.9%	96.3%	95.1%	93.6%	94.3%	94.0%
YOLOv5	95.7%	96.3%	95.9%	92.1%	95.7%	93.8%
ResNeXt + Faster R-CNN	83.8%	88.2%	85.9%	89.7%	92.2%	90.9%
RetinaNet	63.0%	78.1%	69.7%	51.7%	77.2%	61.9%
Resnet + Faster R-CNN	83.8%	88.2%	85.9%	89.7%	92.2%	90.9%

**Table 4:** Evaluation of combined models.

Models	Combined		
	Precision	Recall	F1-Score
Swin-transformer + faster R-CNN	96.5%	97.7%	97.1%
YOLOv5	95.7%	97.1%	96.4%
ResNeXt + Faster R-CNN	92.3%	93.4%	92.9%
RetinaNet	69.7%	82.7%	75.7%
Resnet + faster R-CNN	92.2%	93.4%	92.9%

**Table 5:** Comparison of the precision per LED of two top models.

Species	Swin Transformer		YOLOv5	
	RGB	Fluorescence	RGB	Fluorescence
*Cx. pip*	100.0%	98.2%	96.4%	98.2%
*Cx. tri*	97.3%	95.6%	96.4%	94.8%
*Ae. albo*	98.5%	97.2%	100.0%	91.6%
*Ae. tog*	94.1%	98.1%	96.2%	94.5%
*Ae. vex*	91.1%	100%	95.5%	86.0%
*An.* spp.	98.9%	97.8%	97.8%	90.5%
*Cx. bit*	94.0%	60.4%	70.5%	62.2%
*Cx. ori*	87.1%	100.0%	97.4%	100.0%
*Man. uni*	90.9%	94.2%	100.0%	100.0%
*Oc. kor*	97.7%	93.6%	100.0%	97.7%
*Ar. sub*	85.7%	96.4%	100.0%	100.0%
*Chironomus*	90.9%	92.2%	97.7%	89.0%

## Data Availability

The data presented in this study are not available publicly due to the regulation of funding agency.
